# Do the Activity Indices Used in Axial Spondyloarthritis Capture the Relationships Between Obesity, Smoking and Disease Activity in the Same Way?

**DOI:** 10.3390/jcm13226801

**Published:** 2024-11-12

**Authors:** Rubén Queiro, Sara Alonso-Castro, Ignacio Braña, Marta Loredo, Estefanía Pardo, Stefanie Burger, Valentina Chiminazzo, Mercedes Alperi

**Affiliations:** 1 Rheumatology Division, Central University Hospital of Asturias, 33011 Oviedo, Spain; saraalonsocastro@hotmail.com (S.A.-C.); i.brana.abascal@hotmail.es (I.B.); mloredomart@gmail.com (M.L.); estefaniapardoc@gmail.com (E.P.); stefanie.nam@gmail.com (S.B.); mercedes_alperi@hotmail.com (M.A.); 2 Department of Medicine, Oviedo University School of Medicine, 33006 Oviedo, Spain; 3 Translational Immunology Division, Health Research Institute of the Principality of Asturias (ISPA), 33011 Oviedo, Spain; 4 Rheumatology & ISPA Translational Immunology Division, Hospital Universitario Central de Asturias, Avenida de Roma, S/N, 33011 Oviedo, Spain; 5 Biostatistics Platform, Health Research Institute of the Principality of Asturias (ISPA), 33011 Oviedo, Spain; bioestadistica@ispasturias.es

**Keywords:** obesity, smoking, ASDAS, BASDAI, axial spondyloarthritis, disease activity

## Abstract

**Background/Objectives:** Obesity and smoking have been related to increased disease activity in axial spondyloarthritis (axSpA), but these associations might vary depending on the composite index chosen to assess disease activity. We aimed to check this possibility. **Methods:** Three hundred and thirty consecutive patients were recruited from the monographic axSpA unit of a university center. To assess disease activity, BASDAI and ASDAS-CRP measurements were collected. The factors associated with the different disease activity thresholds of these instruments were analyzed using univariate and multivariate logistic regression models. **Results:** This study included 127 women and 203 men, with a mean age of 47.6 (SD 12.9) years, median disease duration of 8 years [IQR: 4–16], and 63% on biologic therapies. Most patients met the therapeutic goals, with a BASDAI < 4 in 187 (56.7%) and ASDAS inactive/low category in 182 (55.2%). Being male was associated with BASDAI remission (OR 2.63), but smoking reduced this likelihood (OR 0.28). Similar findings were found for ASDAS inactive disease (male: OR 2.09; smoking: OR 0.39). The variables associated with BASDAI ≥ 4 in the multivariate logistic model were the male gender (OR 0.36), age (OR 1.02), smoking (OR 2.39), and obesity (OR 2.94), whereas those associated with active/very active ASDAS categories were the male gender (OR 0.49), age (OR 1.02), and smoking (OR 2.34). However, obesity was not associated with these higher ASDAS categories (*p* = 0.183). **Conclusions:** While the association between smoking and increased disease activity was consistent across all composite activity indices, the obesity–activity relationship was only apparent through the BASDAI.

## 1. Introduction

Spondyloarthritis (SpA) is a group of chronic inflammatory processes with a predilection for the axial skeleton and a common pathogenic and genetic architecture. Within the predominantly axial forms, radiographic forms (the former ankylosing spondylitis) and non-radiographic forms are distinguished, but both entail a serious functional impairment and a decrease in the quality of life for affected patients [1].

Although there are different means of approaching the estimation of the activity and impact that these diseases generate, the two most commonly used composite indices to assess disease activity are the Bath Ankylosing Spondylitis Disease Activity Index (BASDAI) and the Ankylosing Spondylitis Disease Activity Score (ASDAS), the latter being a derivation of the former that incorporates objective parameters such as C-reactive protein (CRP) or the erythrocyte sedimentation rate (ESR) [2]. Currently, the Assessment of Spondyloarthritis International Society (ASAS) group recommends the ASDAS-CRP for the estimation of activity, the monitoring of the response to treatment, and the selection of patients for advanced therapies [3].

Cardiometabolic comorbidity is a common companion of SpA, but there are differences in the prevalence of these factors across different SpA phenotypes. Thus, for example, traditional cardiometabolic risk factors tend to be more prevalent in patients with psoriatic arthritis (PsA) than in axial SpA (axSpA), with the exception of smoking, which is more much prevalent in the latter [4,5,6,7,8,9,10]. In a recent systematic literature review (SLR) with a meta-analysis of axSpA studies, the most prevalent individual comorbidities were hypertension (pooled prevalence 23%), hyperlipidemia (17%), and obesity (14%). Eleven studies consistently showed a higher prevalence of comorbidities in axSpA than controls, and particularly large differences were seen for depression (OR 1.80) and heart failure (OR 1.84). Comorbidities were also associated with axSpA disease activity, functional impairment, quality of life, structural damage, work productivity, and mortality [9]. In this sense, the two factors most consistently linked to these adverse outcomes are obesity and smoking.

However, one aspect that has not been fully clarified in the literature in recent years is whether the association between these factors (mainly tobacco and obesity) and greater inflammatory activity is partially skewed by the particular instrument used to estimate such activity. In fact, some aspects included in the BASDAI, such as fatigue and entheseal pain, are not included in the ASDAS, and it is known that obese patients may report more fatigue and pain than non-obese patients [11,12]. Although there is usually a direct link between fatigue, pain, and inflammation in SpA, fatigue and pain may be multifactorial disease domains not necessarily associated with inflammatory activity in all circumstances [13,14]. Therefore, given the existence of differences in the individual components that constitute each instrument for the measurement of SpA disease activity, it is plausible that factors such as obesity or tobacco may show correlations with greater disease activity when such a link is measured with one instrument; however, no such association may be found when the measurement tool chosen is different. In an attempt to elucidate these assumptions, the present study, which included a significant number of patients with axSpA, was approached with these premises in mind.

## 2. Materials and Methods

### 2.1. Study Population

For the purposes of this observational cross-sectional study, 330 consecutive patients who met the ASAS criteria for axSpA were included [15]. Patients were recruited from the monographic axSpA unit of a university center in Northwestern Spain (total population of one million inhabitants). All patients were of both sexes and over 18 years old. The recruitment period extended from January to October 2023. These patients are seen every 3 months when they start systemic therapy or when they have poor symptomatic control or every 6 months when they progress satisfactorily, reaching the treatment goals (ASDAS remission or low disease activity). The baseline and follow-up protocol are the same for all patients. The work procedures of this SpA unit are regularly audited and currently have the advanced quality seal awarded by the Spanish Society for Healthcare Quality. All patients were informed of the objectives of the study and all signed an informed consent form to participate. This study was conducted in full conformance with the Spanish SAS Order/3470/2009 of the Ministry of Health and Social Policy, local laws and regulations, and the ethical principles laid down in the Declaration of Helsinki. Compliance with the provisions of the new Regulation (EU) 2016/679 of the European Parliament and the Council of 27 April 2016 on Data Protection (GRDP) was also ensured.

### 2.2. Study Variables

Sociodemographic aspects, lifestyles, and previous medical histories were recorded. The following variables were also collected: age at onset, symptom duration, family history of disease, comorbid factors (including components of metabolic syndrome, such as diabetes, hypertension, dyslipidemia, and obesity), axial and peripheral clinical manifestations, number of painful joints, number of inflamed joints, enthesitis, dactylitis, co-manifestations (uveitis, psoriasis, inflammatory bowel disease). The standard disease metrology included the following: disease activity according to the BASDAI and the ASDAS-CRP, physical function according to the Bath Ankylosing Spondylitis Functional Index (BASFI), and the general disease impact according to the ASAS Health Index (ASAS HI) questionnaire. We have previously contrasted the validity, correlations, and feasibility of all these standard measures in our clinical setting [16,17,18]. Pelvis radiographs were performed in anteroposterior projection, as well as anteroposterior and lateral views of the cervical and lumbar spine. The degree of involvement of the sacroiliac joints was assessed by the New York (NY) criteria [19]. However, no specific method of structural damage reading was chosen, while the imaging features of axSpA were simply recorded (sacroiliitis, syndesmophytes, vertebral squaring). Current and past medication in relation to axSpA (NSAIDs, conventional DMARDs, biologic and targeted-specific DMARDs) and the pertinent analytical parameters, such as HLA-B27 (only at baseline), ESR, and CRP, were also collected.

### 2.3. Statistical Methodology

A descriptive statistical study of all the variables was performed, using central and dispersion measures for the quantitative variables, as well as absolute and relative frequencies for the qualitative ones. Variables were compared by parametric and non-parametric statistical tests depending on whether their distribution was normal or not. Comparisons within the study population based on sex (males vs. females) and on the presence of HLA B27 (positive vs. negative) were also included. For the purposes of this study, we used the following cut-off thresholds: BASDAI remission (≤2), active BASDAI (≥4), ASDAS inactive disease (<1.3), and ASDAS high/very high disease activity (≥2.1). To estimate the determinants of the BASDAI remission, active BASDAI, ASDAS inactive disease, and high/very high ASDAS activity categories, univariate and multivariate logistic regression models were used. The strength of the associations between the variables included in the regression models and the different clinical activity thresholds was measured by odds ratio (OR) values with their 95% confidence intervals (CI). The threshold for statistical significance was set at *p* < 0.05. Data were analyzed using the R software (4.3.1 “Beagle Scouts”).

## 3. Results

### 3.1. Summary of Study Population

A total of 127 (38.5%) women and 203 (61.5%) men were included, with a mean age of 47.6 (SD 12.9) years and median disease duration of 8 years [IQR 4–16]. At the time of inclusion, 209 (63.3%) patients were receiving biological therapies, mostly anti-TNF. Most patients were under acceptable disease control, with a mean BASDAI of 3.64 (SD 2.43) and a mean ASDAS of 2.07 (SD 0.852). HLA-B27 determination was positive in 227 (70.7%) patients. Most patients had radiographic axSpA (80%), with a 2.6:1 ratio favoring men. Table 1 summarizes the characteristics of the study population.

### 3.2. Differences Based on Sex

Men showed a significantly longer disease duration [median 10 (IQR 5–20) years] than women [median 6 (IQR 3–12) years], *p* < 0.001. The female population had significantly more active disease according to the BASDAI (4.37 ± 2.41 vs. 3.18 ± 2.33, *p* < 0.001) and ASDAS (2.25 ± 0.78 vs. 1.97 ± 0.88, *p* < 0.001). They also showed poorer functioning according to the BASFI (3.52 ± 2.36 vs. 2.98 ± 2.45, *p* = 0.031). In contrast, men had a higher frequency of advanced bilateral sacroiliitis (93% vs. 77%, *p* < 0.001) and a higher frequency of syndesmophytes (37% vs. 14%, *p* < 0.001). Regarding the analytical determinations, women had a higher ESR [median 8 (IQR 3–17) vs. 4 (IQR 2–8), *p* < 0.001]. A trend bordering on statistical significance was observed in the distribution of HLA-B27 (men: 74.6% vs. women: 64.5%, *p* = 0.07).

### 3.3. Differences Based on HLA-B27

At the time of inclusion, patients with HLA-B27 were younger (46.3 ± 13.1 years vs. 49.9 ± 11.8 years, *p* = 0.016) and had a longer disease duration [median 9.50 (IQR 4–19.3) years vs. 6 (IQR 3–11) years, *p* = 0.003]. The percentage of NSAID users was higher among HLA-B27-positive individuals (75.3% vs. 61.7%, *p* = 0.02). Regarding extra-musculoskeletal manifestations, HLA-B27-positive patients had a higher prevalence of uveitis (19.4% vs. 6.4%, *p* = 0.006), but HLA-B27-negative patients had more inflammatory bowel disease (21.3% vs. 5.3%, *p* < 0.001). HLA-B27-negative subjects had significantly higher mean BASDAI (4.26 ± 2.42 vs. 3.45 ± 2.39, *p* = 0.006) and ASDAS values (2.21 ± 0.81 vs. 2.03 ± 0.87, *p* = 0.035) than B27-positive subjects. Regarding the distribution of cardiometabolic factors, HLA-B27 patients showed a lower diabetes prevalence (2.6% vs. 7.4%, *p* = 0.06). The prevalence of bilateral sacroiliitis was higher among HLA-B27-positive patients (92% vs. 74.5%, *p* < 0.001).

### 3.4. Factors Associated with BASDAI Remission (≤2)

In the univariate logistic regression model, BASDAI remission was significantly associated with being male (OR 2.34, *p* = 0.001), NSAID intake (OR 0.35, *p* < 0.001), smoking (OR 0.33, *p* < 0.001), the ASDAS (OR 0.04. *p* < 0.001), the BASFI (OR 0.39, *p* < 0.001), and HLA-B27 positivity (OR 2.04, *p* = 0.017). In the multivariate logistic regression model, the associated factors were being male [OR 2.63, 95% CI: 1.45–4.91, *p* = 0.002] and smoking [OR 0.28, 95% CI: 0.15–0.53, *p* < 0.0001].

### 3.5. Factors Associated with ASDAS Inactive Disease (<1.3)

In the univariate logistic regression model, the ASDAS inactive disease category was significantly associated with being male (OR 1.99, *p* = 0.03), age (OR 0.97, *p* = 0.022), NSAIDs (OR 0.22, *p* < 0.001), smoking (OR 0.40, *p* = 0.008), the BASDAI (OR 0.22, *p* < 0.001), the BASFI (OR 0.26, *p* < 0.001), CRP (OR 0.70, *p* = 0.031), and sacroiliitis (OR 3.41, *p* = 0.047). The associated factors in the multivariate model were being male [OR 2.1, 95% CI: 1.07–4.26, *p* = 0.036] and smoking [OR 0.39, 95% CI: 0.18–0.79, *p* = 0.012].

### 3.6. Factors Associated with Active BASDAI (≥4)

In the univariate model, the associated variables were the male gender (OR 0.40, *p* < 0.001), NSAIDs (OR 1.85, *p* = 0.015), uveitis (OR 0.43, *p* = 0.011), obesity (OR 2.3, *p* = 0.017), smoking (OR 2.04, *p* = 0.002), the ASDAS (OR 20.4, *p* < 0.001), the BASFI (OR 2.8, *p* < 0.001), the ESR (OR 1.03, *p* = 0.021), HLA-B27 positivity (OR 0.49, *p* = 0.004), and sacroiliitis (OR 0.45, *p* = 0.016). Significant factors in the multivariate regression were being male [OR 0.36, 95% CI: 0.21–0.62, *p* < 0.0001], age [OR 1.03, 95% CI: 1.00–1.05, *p* = 0.028], smoking [OR 2.39, 95% CI: 1.40–4.12, *p* = 0.001], and obesity [OR 2.94, 95% CI: 1.26–7.16, *p* = 0.014]. Table 2 displays the full data included in the univariate and multivariate models.

### 3.7. Factors Associated with High/Very High ASDAS (≥2.1)

In the univariate logistic model, the following significant relationships were found: male gender (OR 0.48, *p* = 0.001), university degree (OR 0.49, *p* = 0.022), NSAIDs (OR 2.6, *p* < 0.001), uveitis (OR 0.51, *p* = 0.04), smoking (OR 2.2, *p* = 0.001), BASDAI (OR 2.6, *p* < 0.001), BASFI (OR 2.2, *p* < 0.001), ESR (OR 1.04, *p* = 0.001), CRP (OR 1.15, *p* = 0.002), HLA-B27 positivity (OR 0.57, *p* = 0.021). In the multivariate model, the following variables stood out: male gender [OR 0.49, 95% CI: 0.29–0.83, *p* = 0.008], age [OR 1.02, 95% CI: 1.00–1.05, *p* = 0.039], and smoking [OR 2.34, 95% CI: 1.39–3.98, *p* = 0.001]. Table 3 shows the full data included in the univariate and multivariate models.

## 4. Discussion

In the present observational study, which included a large sample of patients treated in the axSpA monographic unit from a university center, we have confirmed some classic findings, such as the fact that men are more likely to achieve stringent treatment goals such as BASDAI remission and ASDAS inactive disease, or the fact that smoking significantly hinders the achievement of these goals. In addition, both findings were independent of the exposure to biological therapies in this study. On the other hand, both smoking (OR 2.39) and obesity (OR 2.94) were associated with BASDAI active disease, while this association was only maintained for the first of these factors (OR 2.34) when the activity assessment tool used was the ASDAS, with such a link disappearing in the case of obesity.

Our observations seem to strongly support the role of tobacco in terms of increased inflammatory activity in axSpA. In fact, we did not only find that tobacco hampers the achievement of treatment goals such as inactive disease according to the ASDAS or remission according to the BASDAI, but it was significantly associated with the possibility of active or very active disease according to both measurement instruments. On the other hand, we also found an association between active smoking and structural damage with an OR of 1.9 (95% CI: 1.01–3.74, *p* < 0.05) for syndesmophyte formation. All of this has also been clearly reflected by other studies [20,21,22]. For example, in a meta-analysis of axSpA cross-sectional studies, ever smokers had significantly higher BASDAI scores, BASFI scores, and spinal pain and higher impacts on quality of life than never smokers [21]. Moreover, in another large survey, respondents with PsA and ever smokers reported poorer quality of life, global health, pain, and fatigue than never smokers [22]. The strength of this relationship is also supported by the fact that the strong link between inflammatory activity and smoking is captured regardless of the instrument used to measure disease activity, as confirmed here.

In the SLR with a meta-analysis by Zhao et al., the top five most prevalent comorbidities found in axSpA were hypertension (22.3%), any infection (18.3%), hyperlipidemia (17.1%), obesity (13.5%), and any cardiovascular disease (CVD, 12.3%). The highest obesity prevalence rates were around 27% [9]. In any case, the pooled prevalence was around 14%, i.e., not far from the prevalence of obesity found by us. However, the story regarding the activity–obesity relationship in axSpA is somewhat different when compared to the same relationship for smoking. Thus, although some studies, including meta-analyses, point to a positive correlation between a higher BMI and increased disease activity and other adverse outcomes, not all studies suggest the same [4,5,6,7,8,9,10,23,24,25]. In fact, the distribution of this comorbidity is very different across different SpA phenotypes. In this context, the relationship between obesity and the risk of PsA is very strongly supported, and, currently, obesity is considered one of the key factors in the long-term risk for the development of PsA among psoriasis patients [26]. Furthermore, the prevalence of obesity in PsA is significantly higher than that observed in axSpA [6,12]. Nonetheless, the mechanisms through which obesity or overweight could influence disease activity in axSpA are manifold. First, adipose tissue is not only an energy reservoir but also the source of a host of cytokines and proinflammatory mediators that determine a chronic low-grade inflammatory state [27]. Moreover, many of these proinflammatory pathways are also shared with SpA [27]. Second, according to the pathogenetic theory of the entheseal organ, the mechanical overload caused by overweight and obesity may be the starting point for a mechanical stress-linked aberrant immune response driven by enthesis-resident innate immune lineages [10,28]. These responses, under certain genetic conditioning, can become chronic, giving rise to an overactivated osteo-reparative mechanism in the bone and entheses [10]. Finally, the increased risk of developing osteoarthritis among obese subjects [29], or even other much less studied factors, such as the interplay between breastfeeding, gut dysbiosis, obesity, and the disease [30], would add to an extremely complex network of interactions when it comes to interpreting the effect of obesity on the composite indices designed to assess disease activity.

Given the above, it is not surprising to find disparate associative lines in the different studies published so far that analyze whether or not obese patients with axSpA have a higher inflammatory burden, which in turn could translate into higher values for the composite activity indices. To resolve this issue, Ortolan et al. performed a systematic review and meta-analysis to investigate whether overweight and obesity were associated with higher scores in the BASDAI and ASDAS in axSpA adult patients. A random-effects meta-analysis was used to pool the results, which were expressed as the mean difference (MD) in the BASDAI and ASDAS across BMI groups. The MD in the BASDAI between those with a normal BMI and obese patients was −0.78 (*p* < 0.0001). For the ASDAS, the MD between those with a normal BMI and obesity was −0.42 (*p* < 0.0001). The authors concluded that the differences in terms of increased activity were only significant when comparing obese patients against normal-weight patients, with the effect size of such differences being much more marked with the BASDAI than with the ASDAS [31]. In our study, despite the substantial agreement between both indices (weighted kappa 0.65, Table 1), obese patients were almost three times more likely to score a high BASDAI, while no relationship with the higher ASDAS categories was found. Taking this into account, along with the findings of the aforementioned meta-analysis, it appears that axSpA obese patients tend to score higher on the BASDAI compared to the ASDAS.

The reasons that obese patients score higher on the BASDAI than on the ASDAS remain speculative. The first aspect to consider is that the BASDAI is a six-item instrument that is completely subjective, without any weighting by item, while the ASDAS is a tool derived from the former but formulated logarithmically for a different weighting depending on the item, and with objective elements such as CRP and the ESR [2]. Second, the lack of correctors for each item, together with the potential circularity between some of them, could lead to overestimations of the activity when the tool chosen for this purpose is the BASDAI. On the other side, the ASDAS combines five disease activity variables with only partial overlap, resulting in one single score with better truth (validity), an enhanced discriminative capacity, and improved sensitivity to change as compared to single-item variables [2]. Third, the BASDAI contains two items (fatigue and pain over enthesis points) not present in the ASDAS. We should consider that obese patients may experience fatigue and/or entheseal pain due to non-inflammatory causes, which may thus overestimate the overall BASDAI scoring. For example, in the study by Bindesbøll et al., obese patients reported significantly higher scores on all six questions of the BASDAI scale compared to normal or underweight patients. However, the item with the highest scoring was the one referring to fatigue, with a mean of 6.64 out of 10 [32]. Unfortunately, the study’s authors did not employ the ASDAS, thus preventing a comparative analysis. Finally, it should be mentioned that the close correlations between the BMI, pain, fatigue, and the psycho-emotional burden could influence the scoring of the different components of the BASDAI, which could in turn be another source of overestimation in this score [11,33,34]. Nonetheless, the findings implicit in our study have obvious translation into practice, since, in axSpA obese patients, the instrument for the measurement of disease activity should be the ASDAS rather than the BASDAI. Of course, the BASDAI will continue to be a first-rate tool in all other areas relevant to the disease.

The weaknesses of this study are of different forms. The first are those typical of any cross-sectional study, where the directions of the relationships, whether causal or otherwise, are impossible to establish. Secondly, although the prevalence of obesity in our setting seems to be in line with that of other publications, it is also true that these figures have been significantly higher in other reports [35]. Furthermore, although we developed multivariate models to test the factors linked to the different outcomes, it is practically impossible to consider all of the potential confounders in these formulations [36]. It should be remembered that the primary objective of this study was not focused on obesity as a comorbidity but on the factors associated with remission and high activity in axSpA. Finally, our findings have not been characterized based on the different BMI categories, assuming obesity (BMI ≥ 30) as a categorical variable, which may ultimately be a somewhat crude approximation of the relationships between adiposity and inflammation in SpA. Among the strengths, it is worth highlighting the significant number of patients recruited from a unit specializing in this type of disease and subject to periodic quality audits.

## 5. Conclusions

In summary, both smoking and obesity are two factors associated with increased inflammatory activity in axSpA. However, since the obesity–activity relationship was only evident through the BASDAI in our study, and this is a highly subjective instrument, the factors governing the association between smoking, obesity, and increased axSpA activity could be very different. Therefore, the ASDAS seems a more reliable tool for daily clinical practice in this particular setting.

## Figures and Tables

**Table 1:** Disease characteristics of the study population.

Feature	*n* = 330
Age, yrs, mean (SD)	47.6 (12.9)
Disease duration, yrs, median (IQR)	8 [4,5,6,7,8,9,10,11,12,13,14,15,16]
Men, *n* (%)	203 (61.5)
Women, *n* (%)	127 (38.5)
Radiographic axSpA, *n* (%)	264 (80)
Peripheral involvement, *n* (%)	60 (18.1)
SpA family history, *n* (%)	82 (24.8)
Educational level, *n* (%)	
Primary	87 (26.4)
Secondary	157 (47.6)
University	86 (26.1)
Smokers, *n* (%)	115 (34.8)
Obesity, BMI ≥ 30, *n* (%)	40 (12.1)
SpA-related conditions, *n* (%)	
Psoriasis	25 (7.6)
Enthesitis	33 (10)
Anterior uveitis	56 (17)
Inflammatory bowel disease	34 (10.3)
Lab. variables	
ESR (median, IQR), mm/h	5 [2–10.8]
CRP (median, IQR), mg/dl	0.30 [0.10–1.10]
HLA-B27, *n* (%)	227 (70.7)
Treatment	
NSAIDs, *n* (%)	233 (70.6)
Conventional DMARDs, *n* (%)	57 (17.3)
Oral glucocorticoids, *n* (%)	16 (4.8)
Biological DMARDs, *n* (%)	209 (63.3)
Composite indices	
BASDAI, mean ± SD	3.64 (2.43)
BASFI, mean ± SD	3.19 (2.43)
ASDAS-CRP, mean ± SD	2.07 (0.85)
ASAS-HI, mean ± SD, *n*: 200	5.39 (4.0)
Outcomes *	
BASDAI remission (≤2), *n* (%).	99 (30)
Active BASDAI (≥4), *n* (%)	143 (43.3)
ASDAS inactive disease (<1.3), *n* (%)	70 (21.3)
ASDAS high (≥2.1–3.5), *n* (%)	129 (39.2)
ASDAS very high (>3.5), *n* (%)	18 (5.5)
ASAS HI high impact (>5), *n*: 200 (%)	108 (54)

yrs: years; SD: standard deviation; axSpA: axial spondyloarthritis; BMI: body mass index; ESR: erythrocyte sedimentation rate; CRP: C-reactive protein; HLA: human leukocyte antigen; NSAIDs: non-steroidal anti-inflammatory drugs; DMARDs: disease-modifying anti-rheumatic drugs; BASDAI: Bath Ankylosing Spondylitis Disease Activity Index; BASFI: Bath Ankylosing Spondylitis Functional Index; ASDAS: Ankylosing Spondylitis Disease Activity Score; ASAS-HI: Assessment of Spondyloarthritis International Society Health Index. * The overall weighted Kappa index between the ASDAS and BASDAI was 0.65 (95%IC: 0.59–0.71), indicating substantial agreement between both indices.

**Table 2:** Full regression model for active BASDAI.

Variable	Univariate Model OR (95% CI)	*p*-Value	Multivariate Model OR (95% CI)	*p*-Value
Male	0.38 (0.24–0.61)	<0.001	**0.36 (0.20–0.61)**	<0.0001
Age	1.01 (0.99–1.03)	0.069	**1.02 (1.00–1.05)**	0.028
Education:				
-Secondary	0.90 (0.53–1.52)	0.703		
-University	0.66 (0.36–1.22)	0.187		
Disease duration	0.97 (0.95–1.00)	0.101	0.98 (0.94–1.01)	0.232
Enthesitis	1.33 (0.60–2.98)	0.476		
NSAID	1.84 (1.12–3.03)	0.015		
bDMARD	1.26 (0.80–1.99)	0.307	1.26 (0.80–1.99)	0.810
csDMARD	1.31 (0.74–2.33)	0.344		
Uveitis	0.42 (0.22–0.82)	0.011		
IBD	2.00 (0.97–4.13)	0.058		
Diabetes	2.02 (0.70–5.83)	0.190		
Hypertension	1.30 (0.70–2.41)	0.403		
Obesity	2.31 (1.16–4.59)	0.017	**2.94 (1.26–7.15)**	0.014
Smoking	2.04 (1.29–3.23)	0.002	**2.39 (1.40–4.12)**	0.001
ASDAS	20.39 (10.64–39.09)	<0.001		
BASFI	2.75 (2.24–3.38)	<0.001		
ESR	1.02 (1.00–1.05)	0.021		
CRP	1.05 (0.98–1.12)	0.135		
HLA-B27	0.49 (0.30–0.79)	0.004	0.58 (0.32–1.04)	0.069
Sacroiliitis	0.44 (0.23–0.85)	0.016		
Syndesmophytes	1.17 (0.72–1.90)	0.505		

BASDAI: Bath Ankylosing Spondylitis Disease Activity Score; OR: odds ratio; CI: confidence interval; NSAID: non-steroidal anti-inflammatory drugs; bDMARDs: biological disease-modifying anti-rheumatic drugs; csDMARDs: conventional synthetic disease-modifying anti-rheumatic drugs; IBD: inflammatory bowel disease; ASDAS: Ankylosing Spondylitis Disease Activity Score; BASFI: Bath Ankylosing Spondylitis Functional Index; ESR: erythrocyte sedimentation rate; CRP: C-reactive protein; HLA: human leukocyte antigen. Significant associations in the multivariate model are highlighted in bold.

**Table 3:** Full regression model for the higher ASDAS categories.

Variable	Univariate Model OR (95% CI)	*p*-Value	Multivariate Model OR (95% CI)	*p*-Value
Male	0.48 (0.30–0.75)	0.001	**0.48 (0.28–0.82)**	0.008
Age	1.01 (0.99–1.03)	0.226	**1.02 (1.00–1.04)**	0.039
Education:				
-Secondary	0.60 (0.36–1.02)	0.062		
-University	0.49 (0.26–0.90)	0.022		
Disease duration	0.98 (0.95–1.00)	0.120	0.97 (0.94–1.00)	0.077
Enthesitis	0.75 (0.33–1.72)	0.507		
NSAID	2.59 (1.56–4.31)	<0.001		
bDMARD	1.45 (0.92–2.29)	0.105	1.37 (0.81–2.32)	0.238
csDMARD	1.23 (0.69–2.18)	0.473		
Uveitis	0.51 (0.27–0.97)	0.040		
IBD	1.55 (0.75–3.20)	0.232		
Diabetes	1.08 (0.38–3.07)	0.874		
Hypertension	0.81 (0.43–1.53)	0.527		
Obesity	1.92 (0.97–3.79)	0.059	1.74 (0.77–4.02)	0.183
Smoking	2.15 (1.35–3.41)	0.001	**2.34 (1.39–3.97)**	0.001
BASDAI	2.60 (2.14–3.17)	<0.001		
BASFI	2.16 (1.84–2.53)	<0.001		
ESR	1.04 (1.01–1.07)	0.001		
CRP	1.14 (1.01–1.07)	0.001		
HLA-B27	0.56 (0.34–0.91)	0.021	0.76 (0.43–1.35)	0.360
Sacroiliitis	0.66 (0.34–1.25)	0.206		
Syndesmophytes	1.09 (0.67–1.76)	0.722		

ASDAS: Ankylosing Spondylitis Disease Activity Score; OR: odds ratio; CI: confidence interval; NSAID: non-steroidal anti-inflammatory drugs; bDMARDs: biological disease-modifying anti-rheumatic drugs; csDMARDs: conventional synthetic disease-modifying anti-rheumatic drugs; IBD: inflammatory bowel disease; BASDAI: Bath Ankylosing Spondylitis Disease Activity Score; BASFI: Bath Ankylosing Spondylitis Functional Index; ESR: erythrocyte sedimentation rate; CRP: C-reactive protein; HLA: human leukocyte antigen. Significant associations in the multivariate model are highlighted in bold.

## Data Availability

The materials and raw data described in the manuscript will be freely available to any researcher without breaching any participant’s confidentiality. To facilitate the revision of the results by other researchers, the patient data are available as an Excel file upon request to the corresponding author.
